# Costunolide Loaded in pH-Responsive Mesoporous Silica Nanoparticles for Increased Stability and an Enhanced Anti-Fibrotic Effect

**DOI:** 10.3390/ph14100951

**Published:** 2021-09-23

**Authors:** Xia Niu, Xiaomei Wang, Bingyu Niu, Yanan Meng, Hongwei He, Yucheng Wang, Guiling Li

**Affiliations:** Institute of Medicinal Biotechnology, Chinese Academy of Medical Science & Peking Union Medical College, Beijing 100050, China; niuxia@imb.pumc.edu.cn (X.N.); wangxiaomei@imb.pumc.edu.cn (X.W.); niubingyu@imb.pumc.edu.cn (B.N.); mengyanan@imb.pumc.edu.cn (Y.M.); hehwei@imb.pumc.edu.cn (H.H.)

**Keywords:** pH-responsive mesoporous silica nanoparticles, costunolide, stability, dissolution rate, anti-liver fibrosis

## Abstract

Liver fibrosis remains a significant public health problem. However, few drugs have yet been validated. Costunolide (COS), as a monomeric component of the traditional Chinese medicinal herb *Saussurea Lappa*, has shown excellent anti-fibrotic efficacy. However, COS displays very poor aqueous solubility and poor stability in gastric juice, which greatly limits its application via an oral administration. To increase the stability, improve the dissolution rate and enhance the anti-liver fibrosis of COS, pH-responsive mesoporous silica nanoparticles (MSNs) were selected as a drug carrier. Methacrylic acid copolymer (MAC) as a pH-sensitive material was used to coat the surface of MSNs. The drug release behavior and anti-liver fibrosis effects of MSNs-COS-MAC were evaluated. The results showed that MSNs-COS-MAC prevented a release in the gastric fluid and enhanced the dissolution rate of COS in the intestinal juice. At half the dose of COS, MSNs-COS-MAC still effectively ameliorated parenchymal necrosis, bile duct proliferation and excessive collagen. MSNs-COS-MAC significantly repressed hepatic fibrogenesis by decreasing the expression of hepatic fibrogenic markers in LX-2 cells and liver tissue. These results suggest that MSNs-COS-MAC shows great promise for anti-liver fibrosis treatment.

## 1. Introduction

Liver fibrosis is an ultimate pathological feature of all forms of chronic hepatic damage. Progressive fibrosis often results in cirrhosis, which is responsible for significant morbidity and mortality worldwide [1,2,3]. Due to the lack of effective therapies, liver fibrosis remains a significant public health problem [4]. Currently, liver transplantation is the only curative approach to end-stage cirrhosis but it is very costly. Fortunately, early-stage hepatic fibrosis shows a reversibility, which attracts a lot of interest from researchers seeking a new therapy [5]. However, few drugs have yet been validated clinically or commercially [6,7,8,9]. Therefore, there is an urgent need to develop and explore drugs that reverse fibrogenesis.

Traditional Chinese medicine is a valuable part of Chinese culture and several monomeric compounds from Chinese herbs are potential therapeutics for liver fibrosis [10,11,12]. Costunolide (COS), as a monomeric component of the traditional Chinese herb *Saussurea Lappa* [13], has shown excellent anti-inflammatory [14] and anti-cancer effects [15,16]. Furthermore, our team has previously demonstrated the anti-fibrotic effect of COS on rats [17]. However, costunolide as a sesquiterpene lactone displays very poor aqueous solubility and poor stability in gastric juice, which greatly limits its application via an oral administration [18,19]. To overcome these drawbacks of COS, a suitable oral delivery system is of great necessity to improve its water solubility and stability in gastric juice, thus enhancing the anti-liver fibrosis effect.

pH-responsive drug delivery has been widely developed to trigger drug release along with a wide range of pH in the body [20,21,22]. Mesoporous silica nanoparticles (MSNs) with a tunable pore size and a high pore volume have great potential as a drug carrier [23,24,25,26,27,28]. To achieve a controlled release, MSNs were coated or modified with a pH-sensitive polymer material [20,29,30]. Methacrylic acid copolymer Type A (MAC), with the trade name Eudragit LI00, shows a pH-dependent release owing to different solubility in a different pH [31]. In acidic gastric juice, MAC forms a dense protective layer that can protect acid-sensitive drugs from stomach damage. MAC was dissolved in the intestines, resulting in the release of the drug [32].

Herein, we report a delivery system of COS encapsulated into pH-responsive mesoporous silica nanoparticles and discuss the dispersion effect, drug release behavior and anti-liver fibrosis on rats. COS was loaded in MSNs and then coated with a pH-responsive MAC. The physicochemical properties such as the particle morphology, pore volume, pore size and drug loading were systematically characterized by transmission electron microscopy (TEM), N_2_ adsorption, differential scanning calorimetry (DSC) and X-ray diffraction (XRD). The in vitro anti-fibrotic effect was explored with human HSC line LX-2 cells. The in vivo anti-fibrotic effect on rats was evaluated by testing liver histology, hepatic functions and the level of profibrotic mediators.

## 2. Results and Discussion

### 2.1. Morphology of MSNs-COS and MSNs-COS-MAC

**Figure 1:**
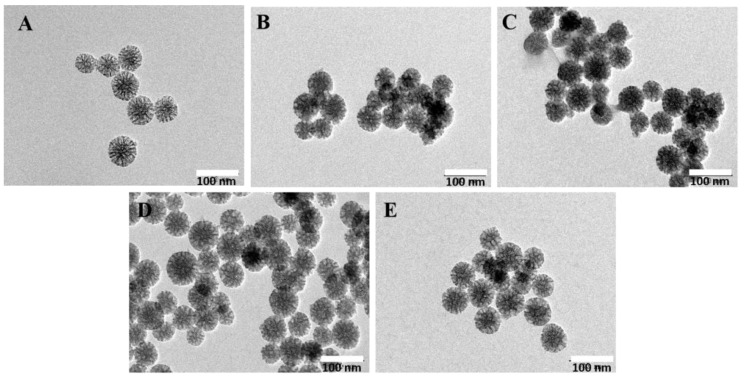
TEM photographs of MSNs (**A**), MSNs-COS (**B**) and MSNs-COS-MAC (**C**). (**D**) MSNs-COS-MAC dispersed in a pH 1.0 hydrochloric acid solution for one hour. (**E**) MSNs-COS-MAC dispersed in distilled water for one hour.

The morphology and particle nanostructures of MSNs, MSNs-COS and MSNs-COS-MAC were studied by TEM. The TEM images showed that the mean pore sizes of MSNs were about 10 nm and the pore channels were clearly visible (Figure 1A). For MSNs-COS and MSNs-COS-MAC, the pore channels were also visible but they were not as clear as in the case of MSNs, indicating that many of the pores were filled with COS and covered by MAC (Figure 1B,C). MSNs and MSNs-COS-MAC were dispersed in distilled water and a pH 1.0 hydrochloric acid solution, respectively. The results showed that there was no obvious difference in appearance between MSNs and MSNs-COS-MAC, indicating MSNs-COS-MAC had a good dispersibility (Figure 1D,E).

### 2.2. The Pore Characteristics of MSNs and MSNs-COS-MAC by the Nitrogen Adsorption–Desorption Method

**Figure 2:**
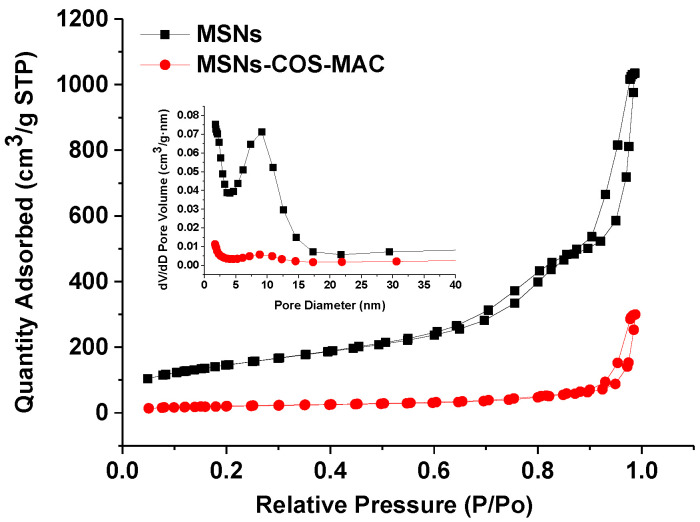
N_2_ adsorption–desorption isotherms and the pore size distribution (inner) of MSNs and MSNs-COS-MAC.

The values of the BET specific surface area (S_BET_), the total pore volume (Vt) and the BJH pore diameter (W_BJH_) of MSNs and MSNs-COS-MAC are presented in Figure 2 and Table 1. It could be seen that MSNs possessed a high S_BET_ (522.5 m^2^/g) and Vt (1.58 cm^3^/g), indicating a great potential to load drugs. For MSNs-COS-MAC, both the surface area and pore volume were clearly reduced, which resulted from the deposition of COS and MAC on the pores. In addition, the shape of the hysteresis loops of MSNs was similar to that of MSNs-COS-MAC, indicating that the drug delivery system was very stable.

### 2.3. Physical State Characterization

**Figure 3:**
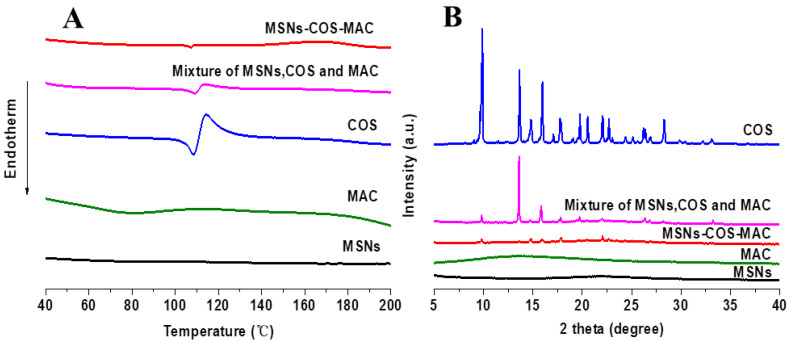
DSC graphs (**A**) and XRD patterns (**B**) of MSNs, MAC, COS, the physical mixture and MSNs-COS-MAC.

The crystalline state of COS, MSNs, MAC, the physical mixture and MSNs-COS-MAC were analyzed by DSC and XRD. In general, a crystalline drug usually exhibits an endothermic melting peak at the melting point [33]. As shown in Figure 3A, a single depression of COS was observed at 107 °C, which was characteristic of its intrinsic melting point. For the physical mixture and MSNs-COS-MAC, this depression was clearly seen at the same temperature, indicating that crystalline COS still existed in MSNs-COS-MAC.

Further confirmation of the crystal properties was achieved using XRD. As shown in Figure 3B, numerous characteristic peaks could be seen in the XRD pattern of the pure crystalline COS that represented the crystalline form. Similar peaks were also observed in the XRD pattern of MSNs-COS-MAC, which indicated that crystalline COS might be still present in MSNs-COS-MAC.

### 2.4. In Vitro Drug Release

**Figure 4:**
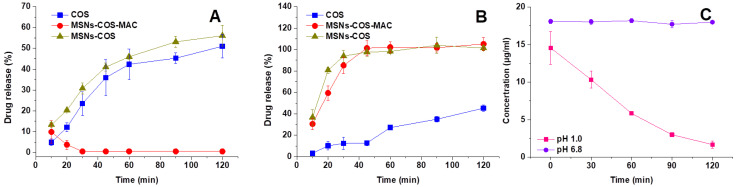
Release profiles of COS from pure COS, MSNs-COS and MSNs-COS-MAC in pH 1.0 hydrochloric acid containing a 0.5% SDS dissolution medium (**A**) and a phosphate buffer of pH 6.8 containing 0.5% SDS (**B**). (**C**) The molecular stability of COS in pH 1.0 hydrochloric acid containing a 0.5% SDS dissolution medium and a phosphate buffer of pH 6.8 containing 0.5% SDS. Each data point represents the mean ± SD of three determinations.

The release profile of COS from pure COS, MSNs-COS and MSNs-COS-MAC in different dissolution media are presented in Figure 4. COS was unstable in pH 1.0 hydrochloric acid and the content reduced to 1.68% ± 0.47% within 2 h (Figure 4C). The results could explain why the observed dissolution rate of the pure COS was quite low in the pH 1.0 dissolution medium containing 0.5% SDS. We speculated that the dissolution of pure COS and MSNs-COS in the pH 1.0 dissolution medium was accompanied by degradation. For MSNs-COS-MAC in the pH 1.0 dissolution medium, COS could be detected in 10 min resulting from an initial burst release, which was attributed to the presence of COS on the surface and near the holes of MSNs. These allowed a certain amount of COS to be released quickly into the release medium. However, COS could be hardly detected after 30 min, probably because MAC coated on the surface of the nanoparticles prevented COS from being released into the dissolution medium. In the pH 6.8 dissolution medium, the dissolution of COS from both MSNs-COS and MSNs-COS-MAC was very fast and almost complete after 45 min whereas the corresponding amounts were only 12.8% ± 2.39 for pure COS. Remarkably, the dissolution rate of COS released from MSNs-COS and MSNs-COS-MAC was faster compared with that from pure COS. As a lipophilic sesquiterpene lactone, COS is poorly water soluble, unstable and easily degradable in gastric fluid. When loaded into MSNs, the dissolution of COS was significantly improved, probably due to the marked dispersing effect of MSNs, which maintain COS into the nanoscales. MSNs-COS-MAC could successfully prevent the degradation of COS passing through the stomach. Thus, it is also possible to reduce the dose of MSNs-COS-MAC administered.

### 2.5. In Vitro Cell Cytotoxicity

**Figure 5:**
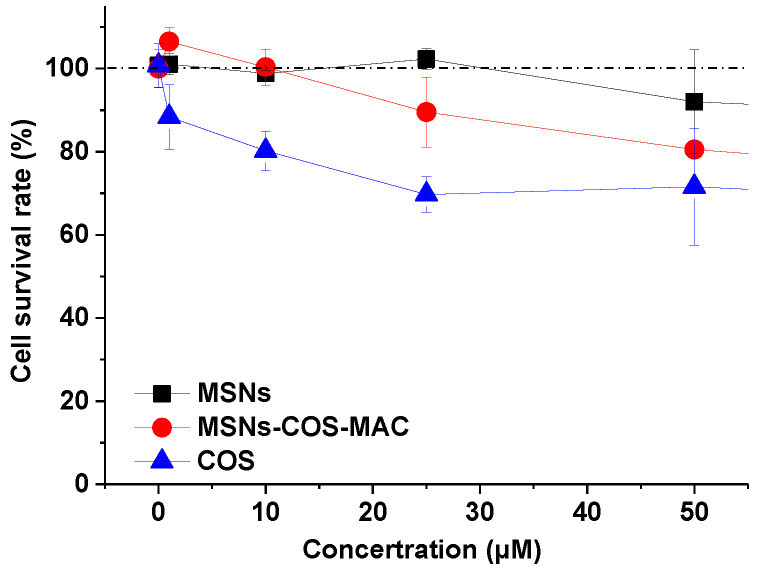
The cell survival rate of LX-2 cells at varying concentrations of pure COS, MSNs and MSNs-COS-MAC. Each data point represents the mean ± SD of three determinations.

Human HSC line LX-2 cells were chosen for in vitro cytotoxicity testing. Figure 5 shows the survival rate of LX-2 cells after a 24 h co-culture with different samples. The tested concentration of MSNs was equivalent to the content of MSNs in the corresponding MSNs-COS-MAC. MSNs showed no cytotoxicity to LX-2 cells. Pure COS exhibited different degree of cytotoxicity at the concentration of 1~50 μM whereas the cell survival rate of MSNs-COS-MAC was higher than that of pure COS at the same concentration. The in vitro cytotoxicity test indicated that MSNs-COS-MAC showed a proper biocompatibility and suitability as a drug delivery system.

### 2.6. MSNs-COS-MAC Significantly Reduce Hepatic Fibrosis-Related Protein Expression in LX-2 Cells and BDL Rats

**Figure 6:**
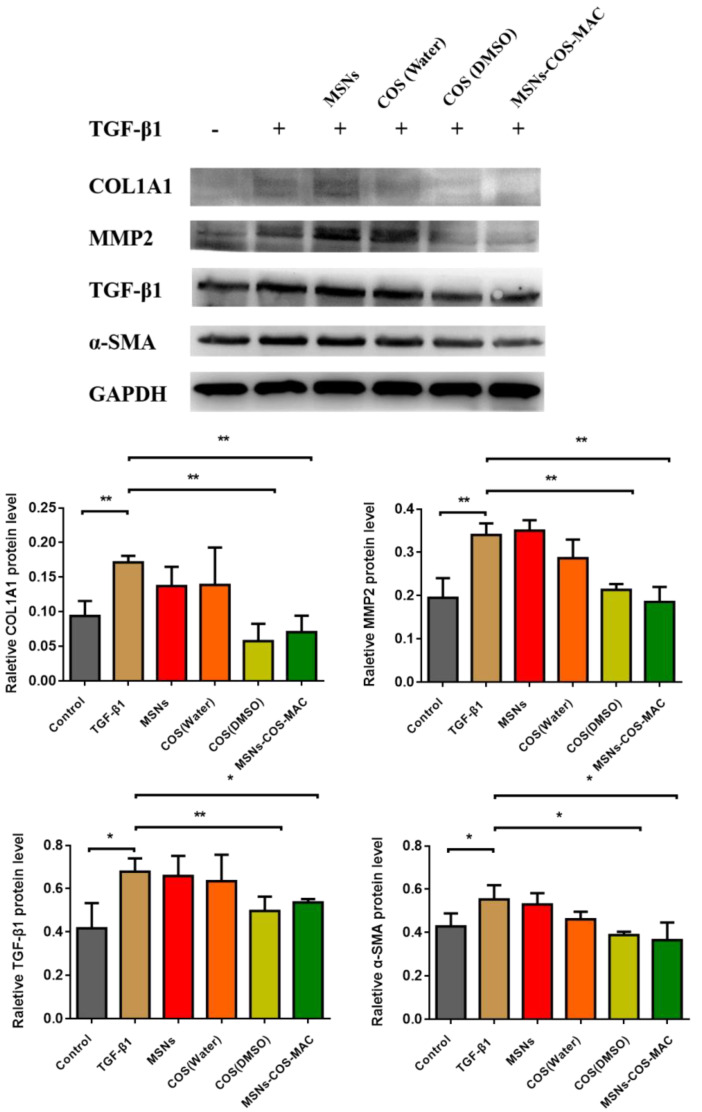
Effects of COS and MSNs-COS-MAC on inhibiting fibrogenetic COL1A1, α-SMA, TGF-β1 and MMP2 protein levels in LX-2 cells. Proteins were extracted and analyzed by a Western blot analysis in the experimental section. The values are expressed as the mean ± SD of triplicate independent experiments (** *p* < 0.01 and * *p* < 0.05 vs. the TGF-β1 group).

Hepatic stellate cells (HSCs) are the major source of fibrogenic myofibroblasts. Activated HSCs are the key cells responsible for collagen deposition and play a central role in hepatic fibrogenesis [34,35]. The fibrogenic markers of the collagen type I α1 chain (COL1A1), transforming growth factor-beta (TGF-β1), α-SMA (α-smooth muscle actin) and MMP2 play an important role in the activation of HSCs [36,37,38]. COL1A1 is a very significant component of the extracellular matrix (ECM). Matrix metalloproteinase-2 (MMP2) contributes to the balance between the synthesis and degradation of ECM [39,40,41]. α-SMA is a biomarker of HSCs. TGF-β1 plays a master role in the activation of HSCs. The neutralization of TGF-β1 activity may be a potentially useful approach for liver fibrosis treatment [42]. In our work, the anti-fibrotic effects of COS and MSNs-COS-MAC were evaluated in vitro by the expression of hepatic fibrogenic markers including COL1A1, α-SMA, TGF-β1 and MMP2 in LX-2 cells using a Western blot analysis. As shown in Figure 6, under the stimulation of TGF-β1 protein (2 ng/mL), the expression of hepatic fibrogenic markers in LX-2 cells was enhanced, which increased the degree of liver fibrosis. After pure COS or MSNs-COS-MAC treatment, MSNs-COS-MAC suspended in sterile water and COS dissolved in DMSO showed a significant decrease in the protein levels of COL1A1, α-SMA, TGF-β1 and MMP2 in LX-2 cells. Interestingly, the anti-liver fibrosis effects of MSNs-COS-MAC were much stronger than those of pure COS when both were suspended in sterile water, indicating MSNs-COS-MAC increased the anti-liver fibrosis effects. Pure COS in sterile water possessed a poor anti-liver fibrosis effect, which may be attributed to poor solubility and far from the concentration of the anti-liver fibrosis effect. Overall, MSNs-COS-MAC increased the in vitro anti-liver fibrosis effect, probably owing to MSNs reducing the crystallite size, increasing the water dispersibility and enhancing the solubility.

**Figure 7:**
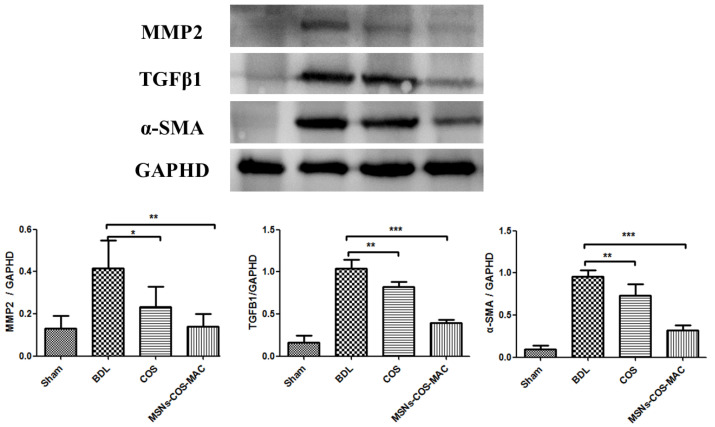
Western blot analysis and semi-quantitation of α-SMA, TGF-β and MMP2 protein levels in liver tissue. The values are expressed as the mean ± SD of four independent experiments. *** *p* < 0.001, ** *p* < 0.01 and * *p* < 0.05, significantly different from the BDL group.

We further studied the effects of COS and MSNs-COS-MAC on the expression of liver fibrosis-related proteins in BDL rats. The results showed that MSNs-COS-MAC and COS significantly lowered the protein expression of these fibrogenic markers. As shown in Figure 7, after the administration of COS (80 mg/kg/day) and MSNs-COS-MAC (40 mg/kg/day) for 14 days, a decreased expression of TGF-β1, MMP2 and α-SMA was observed, which may have been the result of the inhibition of the activation of HSCs. At a half dose of COS, MSNs-COS-MAC showed a stronger anti-fibrotic effect. These findings indicate that MSNs-COS-MAC could inhibit the progression of fibrosis more effectively than COS in BDL rats.

### 2.7. MSNs-COS-MAC Attenuate BDL-Induced Hepatic Fibrosis in Rats

**Figure 8:**
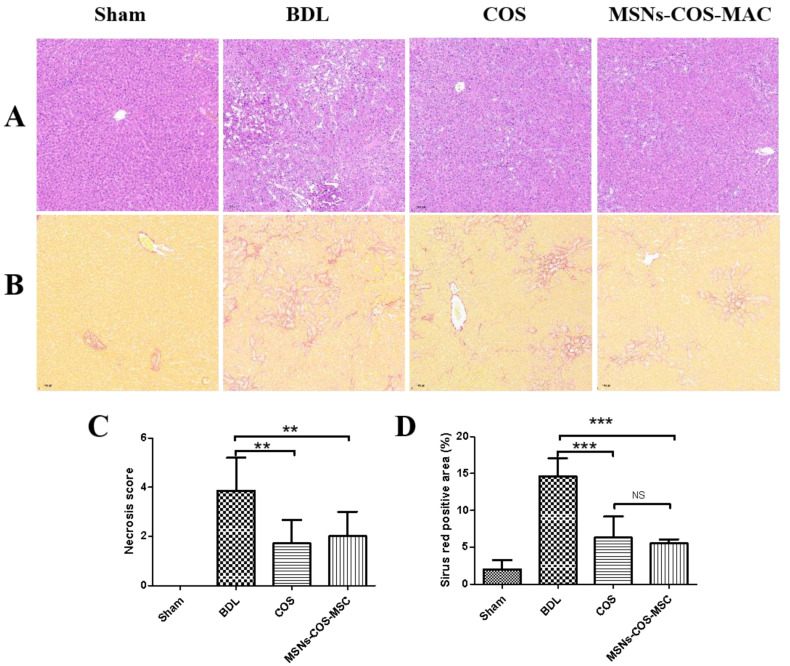
MSNs-COS-MAC improved liver fibrosis in BDL rats. (**A**) Liver pathological changes were detected by H&E staining. (**B**) The degree of liver collagen accumulation was determined by Sirius Red staining. (**C**) A blinded quantitative assessment hepatic necrosis score. (**D**) The percentage of Sirius Red positively stained areas. ** *p* < 0.01 and *** *p* < 0.001, significantly different from the BDL group.

Bile duct ligation (BDL) can induce liver injury and liver fibrosis in rats. Histological examinations through H&E staining (Figure 8A) showed severe cellular damage and necrosis. In addition, Sirius Red staining measured a mass of collagen deposition (Figure 8B). Furthermore, the serum biochemical levels of the BDL group were significantly increased compared with those of the sham group (Table 2). However, these pathological changes were alleviated by COS and MSNs-COS-MAC. The COS group and MSNs-COS-MAC group had a significantly lower necrosis score and smaller Sirius Red-positive stained areas compared with the BDL group (Figure 8C,D). After administration at half the dose of COS, MSNs-COS-MAC still effectively ameliorated parenchymal necrosis, bile duct proliferation and excessive collagen (Figure 8). MSNs-COS-MAC decreased the ALT and AST levels significantly after 14 days of treatment. However, there were no significant changes of ALT and AST levels after COS administration and the GGT level of the COS-BDL group was significantly decreased compared with the BDL group (Table 2). COS treatment at a dose of 80 mg/kg/day showed a weaker effect on the serum biochemical parameters compared with those previous reported [17]. We assumed that the different batches of pure COS may have had a different particle size, which relates to solubility and oral bioavailability [43,44].

## 3. Materials and Methods

### 3.1. Materials

Costunolide (≥98%) was obtained from Nanjing Plant Origin Bio-Technology Co., Ltd. (Nanjing, China). Methacrylic acid copolymer Type A was obtained from Rohm (Germany). Tetraethyl orthosilicate (TEOS) and hexadecyl trimethyl ammonium bromide (CTAB) were purchased from Aladdin (Shanghai, China). 2,2′-Azobis (2-methylpropionamide) dihydrochloride (AIBA, ≥99%) was purchased from Shanghai Yuanye Bio-Technology Co., Ltd. (Shanghai, China). L-lysine was purchased from Rhawn Reagent (Shanghai, China). Recombinant human TGF-β1 protein was obtained from R&D Systems (R&D, USA). Antibodies for α-SMA were obtained from Abcam (Abcam, UK). Antibodies for TGF-β1, MMP2, COL1A1, glyceraldehyde-3-phosphate dehydrogenase (GAPDH) and HRP-conjugated secondary antibodies against mouse or rabbit IgG were purchased from Proteintech (Wuhan, China). A rapid block buffer was obtained from New Cell & Molecular Biotech Co., Ltd (Suzhou, China). All other reagents were of analytical grade and were obtained from commercial sources.

### 3.2. Preparation of MSNs

MSNs were synthesized according to a previously reported method [45]. CTAB was dissolved in deionized water. Octane, TEOS, AIBA, L-lysine and styrene monomer were then added in sequence. The system was kept at 60 °C for 4 h under nitrogen. Afterwards, the resulting product was centrifuged. The precipitate was then purified by ethanol. After centrifugation, the precipitate was removed from the organic template at 600 °C under atmospheric conditions.

### 3.3. Preparation of MSNs-COS-MAC

A total of 5 mL of an ethanol solution of costunolide (76 mg/mL) was dropped into 680 mg of MSNs in an ice bath. The resulting mixture was gently stirred and then the ethanol was evaporated. The residual ethanol was removed by vacuum drying at 4 °C. The obtained powder was called MSNs-COS.

Afterwards, 1 mL of an ethanol solution of MAC (60 mg/mL) was dropped into 350 mg MSNs-COS, gently stirred in an ice bath and then the ethanol was evaporated. The residual ethanol was removed by vacuum drying at 4 °C. The final product was called MSNs-COS-MAC.

### 3.4. Sample Characterization

The morphology of MSNs and MSNs-COS-MAC was observed by a transmission electron microscope (JEM1200EX, JEOL, Japan). The pore characteristics of MSNs and MSNs-COS-MAC were measured using a surface area analyzer (ASAP 2460, micromeritics, USA). The samples were outgassed at 150 °C for 6 h prior to the analysis. The surface areas and the pore diameter distributions were calculated separately according to the Brunauer–Emmett–Teller (BET) theory and the Barrett–Joyner–Halenda (BJH) method. The total pore volumes were determined using the amount adsorbed.

The physical state of MSNs, pure COS, MAC, the physical mixture and MSNs-COS-MAC was evaluated using an X-ray diffractometer (Brucker D8 Advance, Germany) at a scanning speed of 4°/min radiation. Data were obtained from 5° to 40° (diffraction angle 2θ) at a step size of 0.02°.

The DSC profiles of MSNs, pure COS, MAC, the physical mixture and MSNs-COS-MAC were analyzed using a DSC instrument (DSC 1, Mettler, Switzerland). The samples were heated over a temperature range between 40 °C and 200 °C at a rate of 10 °C/min.

### 3.5. Drug Loading by HPLC Analysis

The drug loading of MSNs-COS and MSNs-COS-MAC was determined by HPLC (Nexera-i LC-2040C 3D, Shimadzu, Japan). The chromatographic conditions used in the analysis were as follows: a Shim-pack GIST C18 column (50 mm × 2.1 mm, 2 μm, Shimadzu), a mobile phase of a methanol/pH 2.0 phosphoric acid solution (70:30), a flow rate of 0.3 mL/min, a detection wavelength of 225 nm, a column temperature of 25 °C and an injection volume of 5 μL. COS in the drug-loaded samples was extracted with methanol under an ultrasonic condition and then filtered using a 0.22 μm membrane filter before running the HPLC analysis. Drug loading (%) = (Weight of costunolide in samples /Weight of samples) × 100.

### 3.6. In Vitro Dissolution

The dissolution profiles of pure COS and COS-loaded nanoparticles were determined using a dissolution tester (ZRS-8LD, China) according to the USPII paddle method. A pH 1.0 hydrochloric acid solution with 0.5% SDS and a pH 6.8 phosphate buffer solution with 0.5% SDS were chosen as the dissolution media. A total of 7 mg of pure COS and the drug-loaded samples (equivalent to 7 mg COS) were added to a 900 mL dissolution medium and stirred at 100 rpm at 37 ± 0.5 °C for 120 min. At predetermined time intervals, 5 mL sample solutions of the media were withdrawn and replaced with 5 mL of a fresh dissolution medium to maintain a constant volume. The sample solutions were then filtered using a 0.22 μm membrane before running the HPLC analysis. The condition of HPLC was ready for use before the dissolution experiment. The samples of each time point were immediately measured by HPLC.

### 3.7. In Vitro Cytotoxicity

Human HSC line LX-2 cells (Millipore Cat # SCC064, RRID: CVCL-5792) were chosen for in vitro cytotoxicity testing. The LX-2 cells were cultured in DMEM/GlutaMAX I (Invitrogen, America) with 10% fetal bovine serum and 1% penicillin/streptomycin at 37 °C in an atmosphere of 5% CO_2_. The cell suspension was seeded into 96-well plates at 100 μL per well and incubated for 24 h. MSNs, pure COS and MSNs-COS-MAC suspensions containing different concentrations were then added to 96-well plates at 100 μL per well and incubated for 24 h. A CCK8 solution of 10 μL was then added to the 96-well plates and incubated for 2 h. Finally, the absorbance was determined at 450 nm by an optical microscope (BioTek, SYNERGYH1, America) and the cell survival rate was calculated according to the formula: Cell survival rate (%) = Absorbance of sample/Absorbance of control × 100.

### 3.8. The Anti-Fibrotic Effects In Vitro on Human HSC Line LX-2 Cells

The LX-2 cells were cultured as described above and seeded in 6-well plates. MSNs-COS-MAC was suspended in sterile water. Pure COS was suspended in DMSO and sterile water. Upon reaching 90–95% confluences, the LX-2 cells were starved with a serum-free culture. After 24 h, the cells were treated with TGF-β1 (2 ng/mL) and COS or MSNs-COS-MAC (10 µM) for 12 h. The protein was then extracted with a RIPA buffer. The total protein was then extracted with the RIPA buffer and determined by a BCA protein assay kit (Beyotime Biotechnology, China). The protein samples were separated by 10% SDS-PAGE gel and the resulting protein bands were analyzed on the imager (Tanon5200, China) according to a previously reported method [45].

### 3.9. The Anti-Fibrotic Effects In Vivo on Rats

#### 3.9.1. Bile Duct Ligation (BDL) Surgery in Rats

Sprague Dawley (SD) rats were supplied by HFK Biotechnology Co., Ltd. (Beijing, China). Twenty-eight SD rats (body weight 200 ± 20 g) were randomly assigned into four groups (Sham, BDL, BDL-COS and BDL-MSNs-COS-MAC) followed by a randomization procedure as follows: group I, Sham group (sham operation without BDL as a control group, *n* = 7); group II, BDL group (BDL surgery and no drug administration, *n* = 7); group III, BDL-COS group (BDL surgery and treated with pure COS, 80 mg/kg/day, *n* = 7); group IV, BDL-MSNs-COS-MAC group (BDL surgery and treated with MSNs-COS-MAC, 40 mg/kg/day, *n* = 7). The rats were then anaesthetized with isoflurane and the bile ducts were ligated except in the sham group. Twenty-four hours after surgery, the BDL rats received daily gavages with 80 mg/kg COS suspended and 40 mg/kg MSNs-COS-MAC for 14 days in a 0.5% sodium carboxymethyldellulose solution, respectively. MSNs-COS-MAC was suspended with a 0.5% sodium carboxymethyldellulose solution and then the gavages were administered immediately. The pH of the MSNs-COS-MAC suspension was about 7. Blood samples and liver samples were collected. All samples were stored at –80 °C for further analysis.

#### 3.9.2. Serum Biochemical Parameters

The kits of alanine aminotransferase (ALT), aspartate transaminase (AST), alkaline phosphatase (ALP) and γ-glutamyl transpeptidase (GGT) were purchased from Zhongsheng Beikong Biotechnology (Beijing, China) and then measured with a Hitachi 7100 Analyzer.

#### 3.9.3. Histological Analysis of the Liver Tissue

Liver tissues were stained with Sirius Red and hematoxylin (HE). The hepatic inflammation and bile duct proliferation were quantified in a blinded manner on a 1 to 5 point scale. Sirius Red-stained sections from each animal were observed at a low magnification and analyzed using ImageJ software to calculate the percentage of the hepatic fibrous area.

#### 3.9.4. Western Blot Analysis

The total proteins from the liver tissue were extracted using a RIPA lysate buffer containing a protease inhibitor on ice and then centrifuged (15,000 rpm) at 4 °C for 20 min. The total protein was determined by a BCA protein assay kit (Beyotime Biotechnology, China). The equal amount of protein was applied to 10% SDS-PAGE gel and transferred to a polyvinylidene difluoride (PVDF) membrane (Millipore Corp, Atlanta, GA, US). After being blocked for 10 min with a rapid block buffer, the membrane was then immunoblotted with primary antibodies overnight at 4 °C followed by horseradish peroxidase (HRP)-conjugated secondary antibodies (1:10,000) at room temperature for 2 h. The protein bands were analyzed on the imager (Tanon5200, China).

### 3.10. Statistical Analysis

All quantitative data were presented as mean ± SD with a minimum of three independent samples and analyzed by a one-way analysis of variance (ANOVA). *P*-values of < 0.05 were regarded as statistically significant. The statistical analysis was performed in GraphPad Prism 5.

## 4. Conclusions

We successfully developed MSNs-COS-MAC as a pH-responsive nanoparticle delivery system for COS to enhance the dissolution and reduce the degradation in gastric fluid and ameliorate hepatic fibrosis at a reduced dose. Encapsulated in MSNs-COS-MAC, the crystalline form of COS was not changed. The experimental results suggested that MSNs-COS-MAC exhibited a delayed release in gastric fluid, a rapid dissolution into the intestine juice and a greatly improved anti-hepatic fibrosis effect. In summary, the exploration of MSNs-COS-MAC in this paper provides a good foundation for the development of an anti-fibrotic formulation of COS.

## Figures and Tables

**Table 1:** Surface area, pore volume, pore diameter and degree of drug loading of MSNs and MSNs-COS-MAC.

Samples	S_BET_ (m^2^/g)	Vt (cm^3^/g)	*w*_BJH_ (nm)	Drug Loading (% HPLC)
MSNs	522.5	1.58	11.02	–
MSNs-COS-MAC	72.5	0.46	–	30.5 ± 2.45

**Table 2:** Serum biochemical markers of bile duct ligated (BDL)-treated rats, mean ± SD, *n* = 7.

	Sham	BDL	BDL-COS	BDL-MSNs-COS-MAC
ALT (U L^−1^)	39.00 ± 2.37	105.00 ± 15.98 ^###^	95.86 ± 28.57	66.29 ± 9.72 ***
AST (U L^−1^)	138.50 ± 17.85	540.71.00 ± 78.44 ^###^	486.14 ± 141.70	343.14 ± 116.21 **
ALP (U L^−1^)	168.17 ± 15.43	331.29 ± 67.42 ^###^	331.86 ± 41.72	324.43 ± 103.48
GGT (U L^−1^)	0.17 ± 0.41	47.43 ± 15.34 ^###^	31.43 ± 9.54 *	45.00 ± 25.17

**^###^***p* < 0.001, significantly different from the sham group. * *p* < 0.05, ** *p* < 0.01 and *** *p* < 0.001, significantly different from the BDL group.

## Data Availability

Data are contained within the article.
